# Long‐term cardiovascular effects of vandetanib and pazopanib in normotensive rats

**DOI:** 10.1002/prp2.477

**Published:** 2019-05-31

**Authors:** Samantha L. Cooper, Joanne J. Carter, Julie March, Jeanette Woolard

**Affiliations:** ^1^ Division of Physiology, Pharmacology and Neuroscience School of Life Sciences Queen's Medical Centre University of Nottingham Nottingham UK; ^2^ Centre of Membrane Proteins and Receptors (COMPARE) University of Birmingham and University of Nottingham Midlands UK

**Keywords:** cardiovascular pharmacology, hypertension, pazopanib, radiotelemetry, receptor tyrosine kinase inhibitors, vandetanib

## Abstract

Vandetanib and pazopanib are clinically available, multi‐targeted inhibitors of vascular endothelial growth factor (VEGF) and platelet‐derived growth factor (PDGF) receptor tyrosine kinases. Short‐term VEGF receptor inhibition is associated with hypertension in 15%‐60% of patients, which may limit the use of these anticancer therapies over the longer term. To evaluate the longer‐term cardiovascular implications of treatment, we investigated the “on”‐treatment (21 days) and “off”‐treatment (10 days) effects following daily administration of vandetanib, pazopanib, or vehicle, in conscious rats. Cardiovascular variables were monitored in unrestrained Sprague‐Dawley rats instrumented with radiotelemetric devices. In Study 1, rats were randomly assigned to receive either daily intraperitoneal injections of vehicle (volume 0.5 mL; n = 5) or vandetanib 25 mg/kg/day (volume 0.5 mL; n = 6). In Study 2, rats received either vehicle (volume 0.5 mL; n = 4) or pazopanib 30 mg/kg/day (volume 0.5 mL; n = 7), dosed once every 24 hours for 21 days. All solutions were in 2% Tween, 5% propylene glycol in 0.9% saline solution. Vandetanib caused sustained increases in mean arterial pressure (MAP), systolic blood pressure (SBP), and diastolic blood pressure (DBP) compared to baseline and vehicle. Vandetanib also significantly altered the circadian cycling of MAP, SBP, and DBP. Elevations in SBP were detectable 162 hours after the last dose of vandetanib. Pazopanib also caused increases in MAP, SBP, and DBP. However, compared to vandetanib, these increases were of slower onset and a smaller magnitude. These data suggest that the cardiovascular consequences of vandetanib and pazopanib treatment are sustained, even after prolonged cessation of drug treatment.

## 1 INTRODUCTION

One of the most potent mediators of angiogenesis is vascular endothelial growth factor (VEGF).^1^, ^2^ The VEGF family of dimeric polypeptide ligands includes VEGF‐A, VEGF‐B, VEGF‐C, VEGF‐D, placental growth factor (PlGF), the virus‐encoded VEGF‐E, and the snake venom‐derived VEGF‐F.^2^ VEGF‐A has been identified as the key regulator of angiogenesis, a process critical for tumor progression.^3^, ^4^ As a result, several antiangiogenic therapeutics aimed at targeting VEGF, or its receptors, have emerged as key adjuvant cancer treatments in the prolongation of progression‐free survival and, in some cases, overall survival.^1^

A number of receptor tyrosine kinase inhibitors (RTKIs) that target VEGFR2 5, 6 are now routinely used in the treatment of advanced stage, or metastatic disease.^1^, ^6^, ^7^ Vandetanib (Caprelsa^®^) and pazopanib (Votrient^®^) are used to treat medullary thyroid cancer and advanced renal cell carcinoma, respectively.^8^, ^9^ Furthermore, sunitinib (Sutent^®^) is used for the treatment of renal cell carcinoma, gastrointestinal stromal tumors and pancreatic neuroendocrine tumors, while sorafenib (Nexavar ^®^) is indicated for late‐stage treatment of hepatocellular carcinoma or advanced metastatic renal cell carcinoma.^10^, ^11^

RTKIs that inhibit VEGFR2 tyrosine kinase activity effectively reduce angiogenesis, potentially by decreasing the number of vessel nodes and vessel length in a tumor.^1^, ^4^, ^7^ Over time, this induces a hypoxic environment within the tumor that leads to tumor cell death and a reduction in tumor size.^12^ Since these anticancer therapies target the tumor microvascular environment, it is perhaps not surprising that significant cardiovascular side effects have been reported, both clinically and in preclinical studies.^13^, ^14^ For example, in patients treated with bevacizumab, a humanized monoclonal antibody that targets the VEGF‐A ligand, 28% of patients developed Grade 3 hypertension (>180 mm Hg systolic pressure).^15^ This pressor effect is also observed with small molecules that inhibit VEGFR2 signaling.^15^ Thus, 15%‐60% of patients treated with small molecule RTKIs have been reported to be associated with an increased incidence of hypertension.^16^ Furthermore, the escalation of hypertension in these patients is linked with severe cardiovascular complications such as thromboembolism, intracerebral hemorrhage, stroke, and myocardial infarction, often resulting in termination of treatment.^13^ Vandetanib has been shown to cause ECG prolongation, and hypertension in over 32% of patients, with 9% of those treated with vandetanib developing Grade 3 hypertension.^17^ Similarly, hypertension occurs in approximately 40%‐57% of patients treated with pazopanib, with 3% of those developing Grade 3 hypertension.^18^, ^19^ For most patients, the onset of hypertension occurs within 4 weeks of treatment.^18^, ^19^

We recently recapitulated the hypertensive effects of a number of RTKIs that target VEGFR2^5^ in a conscious rat model^20^ in which the regional hemodynamic effects of four RTKIs (including pazopanib and vandetanib) were monitored over a period of 4 days. This study showed that RTKIs consistently caused hypertension, which was associated with regionally selective vasoconstrictions, particularly in the hindquarters and mesenteric vascular beds.^20^ Short‐term radiotelemetric studies in rats have also examined the effects of RTKIs on blood pressure and heart rate (HR). Isobe et al^21^ dosed animals over four consecutive days with vehicle, cediranib (0.1‐10 mg/kg), sunitinib (0.5‐40 mg/kg), or sorafenib (0.1‐5 mg/kg).^21^ The study clearly demonstrated significantly elevated blood pressures within a few days of the initiation of dosing with RTKIs. Similarly, Lankhorst et al^22^ showed a dose‐dependent elevation in mean arterial pressure (MAP) following administration of sunitinib (7‐26.7 mg/day) over a period of 5 days.^22^ More recently Collins et al^23^ evaluated more selected kinase inhibitors with VEGFR‐2 activity, AZ1, and regorafenib, again showing pressor effects when treatment was continued over a period of 4 days.^23^

In the present study, we have used radiotelemetry to evaluate the longer‐term impact of prolonged RTKI treatment on the cardiovascular system, with particular attention given to the extent to which hypertension is sustained, both during and after cessation of treatment. To this end, we have evaluated the cardiovascular effects of two clinically available RTKIs, vandetanib, and pazopanib, over a 21‐day dosing “on”‐period and during a 10‐day post “off”‐treatment period, in freely moving, telemetered rats.

## 2 MATERIALS AND METHODS

### 2.1 Animals

Adult, male, Sprague‐Dawley rats (Charles River Laboratories, UK) weighing 300‐400 g were housed in groups in a temperature‐controlled (21‐23°C) environment with a 12 hours light–dark cycle (lights on at 06:00) and free access to food (18% Protein Rodent Diet; Teklad Global, Bicester, United Kingdom) and water ad libitum. Following surgery, telemetered rats were pair‐housed in standard individually ventilated cages, placed upon single DSI receivers, with a noninstrumented companion rat throughout the duration of the study. Cages were prepared with bedding material and enrichment. All procedures were carried out with approval of the University of Nottingham Animal Welfare Ethical Review Board under Home Office Project and Personal License Authority. Every effort was made to ensure that animals experienced minimal discomfort. Twenty‐two rats were used for this study, and results are recorded in accordance with the ARRIVE guidelines for reporting experiments involving animals.^24^

### 2.2 Surgical implantation of radiotelemetric devices

Surgery was performed under general anesthesia (fentanyl citrate and medetomidine, 300 μg/kg each, i.p., supplemented as required). Sprague Dawely rats were implanted with telemetry transmitters, DSI C50PXT (Study 1: vandetanib), and DSI HDS11 (Study 2: pazopanib) (Data Sciences International, St. Paul, MN USA). For both devices, the catheter tip was advanced via the distal abdominal aorta until approximately 1 cm below the renal artery, as described previously.^25^ The positive ECG lead was secured to the xiphoid sternum and the negative ECG lead was placed over the manubrium and tunneled subcutaneously from the abdomen to the anterior of the neck.^26^ The telemetry device was secured to the body wall. Following surgery, the animals received reversal of anesthesia and postoperative analgesia provided by atipamezole hydrochloride (1 mg/kg, s.c.) and buprenorphine (0.02 mg/kg, s.c.). A second dose of buprenorphine (0.02 mg/kg, i.p.) was given as an analgesic 4 hours after surgery and a daily dose of buprenorphine (30 mg/kg, s.c.) and carprofen (0.5%) was administered for 4 days postsurgery. The animals were recovered for at least 10 days, and after a satisfactory inspection from the Named Veterinary Surgeon the animals were randomized to either a vehicle or treatment group. At the end of each experiment, rats were euthanized via a schedule 1 method, with Euthatal (60‐80 mg, i.p.) and exsanguination.

### 2.3 Telemetry

Following recovery from surgery, radiotelemetry devices were switched on and baseline recordings of HR, mean systolic blood pressure (SBP), mean diastolic blood pressure (DBP), and mean arterial blood pressure (MAP) were recorded every 15 minutes for a period of 1 min throughout the experimental period (approximately 35 days in total). Baseline recordings were collected for a minimum of 4 days prior to the start of treatment. After this baseline monitoring period, rats were randomly assigned into two studies:

*Study 1:* Rats were randomly administered vehicle (volume 0.5 mL; n = 5) or vandetanib 25 mg/kg/day (volume 0.5 mL; n = 6), dosed i.p, once every 24 hours for 21 days. All solutions were prepared in (2% Tween, 5% propylene glycol in 0.9% saline solution).

*Study 2:* Animals were randomly assigned to receive vehicle (volume 0.5 mL; n = 4) or pazopanib 30 mg/kg/day (volume 0.5 mL; n = 7), dosed i.p, once every 24 hours for 21 days. All solutions were prepared in (2% Tween, 5% propylene glycol in 0.9% saline solution).

### 2.4 Drugs, chemical reagents, and other materials

Pazopanib and vandetanib were purchased from Sequoia Research Products, UK. Fentanyl citrate was purchased from Jansen‐Cilac Ltd, UK. Medetomidine (Domitor), carprofen (Rimadyl) and atipamezole hydrochloride (Antisedan) were purchased from Pfizer, UK. Buprenorphine (Vetergesic) and pentobarbitone (Euthatal) were purchased from Alstoe Animal Health, UK. Tween and propylene glycol were purchased from Sigma‐Aldrich, UK.

### 2.5 Data analysis

Twenty‐four hours (00:00‐23:45), morning (06:00‐12:00) and evening (18:00‐23:45) recording averages (means) were calculated to give HR, MAP, SBP, and DBP values. Change from baseline calculations (time point ‐ average of the baseline = change from baseline) were used to determine ΔHR, ΔMAP, ΔSBP, and ΔDBP.

To evaluate vandetanib‐ or pazopanib‐induced changes in circadian cycling during initial 2 days of dosing and the last 2 days of dosing followed by the 10‐day postdosing period with vandetanib and pazopanib, each 24 hours day was divided into 6, 3 hours bins (06:00‐09:00, 09:00‐12:00, 12:00‐15:00, 15:00‐18:00, 18:00‐21:00, and 21:00‐24:00) and HR, MAP, SBP, and DBP were calculated for: (1) the last 2 days of baseline (pre‐treatment) and the first 3 days of dosing with either vandetanib, pazopanib, or vehicle; (2) days 20 and 21 of dosing with vandetanib, pazopanib, or vehicle, followed by the 10‐day “off”‐treatment period (days 22‐31).

All data were expressed as mean ± SEM. Data were analyzed using Prism 6 software (GraphPad software, USA). Differences were considered significant if the *P*‐value was less than 0.05. To assess statistical differences between vehicle or treatment groups (vandetanib or pazopanib), two‐tailed comparisons of the integrated area under the curve were made using the Mann‐Whitney‐U test. A repeated measures ANOVA with Sidak's correction was used to compare drug treatment to vehicle at individual time points. A repeated measures nonparametric ANOVA with Dunnet's correction was used to compare each time point to the baseline average.

## 3 RESULTS

In the present study, we investigated hemodynamic responses to vandetanib (25 mg/kg/day, i.p.) or pazopanib (30 mg/kg/day, i.p.) in conscious telemetered rats following chronic treatment, over a 21‐day dosing period and a 10‐day “off”‐treatment period. The circadian rhythm, oscillating cycles of cardiovascular variables over a 24 hours time period, were also measured during the last 2 days of baseline followed by the first 3 days of dosing with vandetanib, pazopanib, or vehicle and the last 2 days of dosing followed by the 10‐day posttreatment period. This was to investigate whether vandetanib or pazopanib interfered with the natural cycling of hemodynamic processes over a 24 hours period and over multiple days. Baseline cardiovascular variables before the administration of RTKI or the corresponding vehicle are shown in Table 1.

**Table 1:** Baseline cardiovascular variables for heart rate (HR), mean arterial pressure (MAP), systolic blood pressure (SBP), and DBP (24 h). Statistics: *P < 0.05; Comparing vehicle and vandetanib groups or vehicle and pazopanib groups. Values have been rounded to the nearest whole number

	HR (beats/min)	MAP (mm Hg)	SBP (mm Hg)	DBP (mm Hg)
Series 1 baseline values				
Vehicle 1 (n = 5)	395 ± 2	108 ± 0	128 ± 0	92 ± 0
Vandetanib 25 mg/kg/day (n = 6)	373 ± 4*	108 ± 0	128 ± 0	92 ± 1
Series 2 baseline values				
Vehicle 2 (n = 4)	370 ± 5	101 ± 0	124 ± 0	83 ± 0
Pazopanib 30 mg/kg/day (n = 7)	372 ± 4	99 ± 0	120 ± 0*	82 ± 0

### 3.1 Cardiovascular effects of Vandetanib

#### 3.1.1 Heart rate

A single, daily dose of vandetanib for 21 days did not significantly alter HR compared to baseline at any time point during either the dosing period or the “off”‐treatment period (Figure S1a,c,e). Vandetanib did, however, significantly decrease the change in HR (ΔHR; relative to the corresponding initial baseline measurement obtained for each animal) on the 4th and 10th day of dosing and the 2nd, 6th, and 10th day of the “off”‐treatment period (Figure S1b). The majority of these ΔHR decreases appeared during the animal's active phase (21:00‐06:00) for both the dosing and the posttreatment periods (Figure S1d). However, declines in HR and ΔHR over time were also found with vehicle (Figures S1a‐f) suggesting that the changes in HR from the initial baseline measurements were probably not entirely due to vandetanib. This is probably a consequence of habituation to the dosing regimens and a reduction in sympathetic nervous system activity.

**Figure 1:**
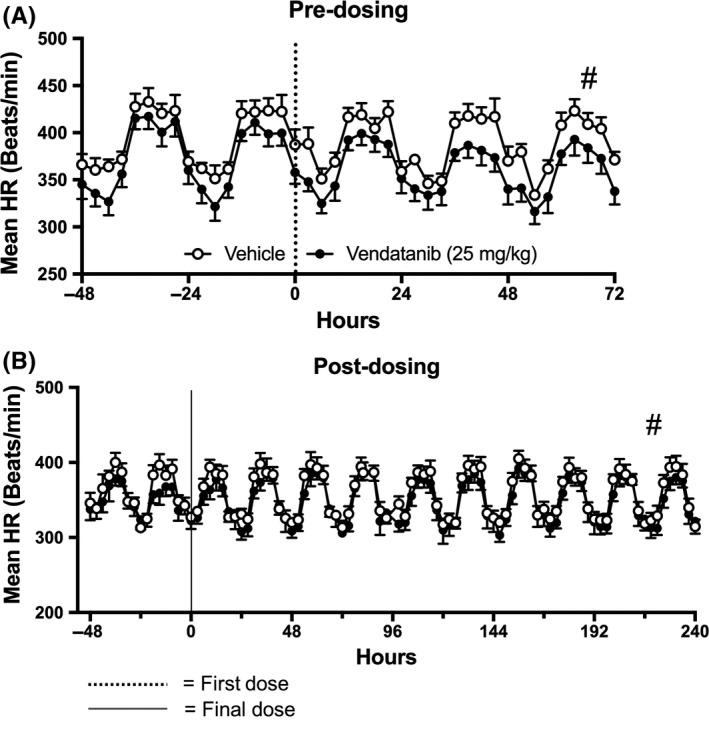
Circadian oscillations of the mean heart rate (HR) of rats dosed with vandetanib 25 mg/kg/day (n = 6) and vehicle (n = 5). During: (A) 2 days prior to dosing followed by the first 3 days of dosing; and (B) the last 2 days of the dosing period followed by 10 days “off‐treatment”. Data are displayed as mean ± SEM. *P < 0.05 comparing each time point to baseline; ^+^ *P* < 0.05 comparing vehicle vs vandetanib at the same time point and ^#^ *P* < 0.05 comparing area over or under the curve of vehicle vs vandetanib

When integrated area under the curve analysis was undertaken, it identified significant differences between the vandetanib and vehicle groups for the duration of the 21‐day dosing period and the 10‐day posttreatment period (Mann‐Whitney U test, # = *P* < 0.05; Figure S1d‐f). Vandetanib did not significantly alter the circadian rhythm of HR compared to baseline during the initial 3 days of dosing (Figure 1A) or the last 2 days of dosing followed by the 10‐day posttreatment period (Figure 1B). Integrated area under the curve analysis of these data detected significant differences between vehicle and vandetanib during the initial 3 days of dosing and the 10‐day posttreatment period (Figures 1A‐B). Thus, the peak‐trough amplitude of the circadian rhythm in HR decreased during the first 3 days of treatment with vandetanib (Figure 1A) and then recovered during the “off”‐treatment period (Figure 1B). Bradycardic tendencies were also observed during the days 1‐5 and 5‐10 averages, of the “off”‐treatment period (Table S1).

#### 3.1.2 Blood pressure

**Figure 2:**
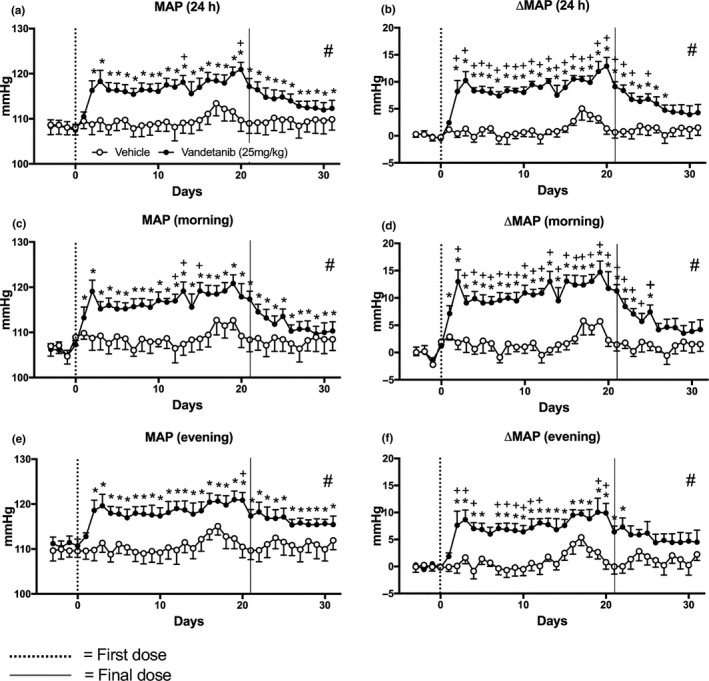
Mean arterial blood pressure (MAP) of rats dosed with vandetanib 25 mg/kg/day (n = 6) and vehicle (n = 5). A, MAP and B, change in MAP compared to baseline (ΔMAP) measured for 24 hours; C, MAP and D, ΔMAP measured during the morning (06:00‐12:00); E, MAP and F, ΔMAP measured during the evening (18:00‐23:45). Data are displayed as mean ± SEM. *P < 0.05 comparing each time point to baseline; ^+^ *P* < 0.05 comparing vehicle vs vandetanib at the same time point and ^#^ *P* < 0.05 comparing area over or under the curve of vehicle vs vandetanib

Significant pressor effects of vandetanib (*P* < 0.05) were observed on both MAP (Figure 2A,C,E) and the change in MAP from the original baseline measurement of each individual animal (ΔMAP) (Figure 2B,D,F). There was a significant increase in ΔMAP compared to baseline from the first active evening phase of vandetanib dosing and this elevation remained throughout the 21‐day dosing period and 6 days into the “off”‐treatment period (Figure 2B,D,F). The maximum response was achieved on the 20th day of treatment (24 hours measurement; vandetanib = +13 ± 2 mm Hg, vehicle = +1 ± 1 mm Hg, *P* < 0.05; Figure 2A). Over the 24 hours recording period, ΔMAP appeared to have a sustained component during the “off”‐treatment period (Figure 2B). This was particularly apparent during the resting morning recordings (06:00‐12:00) (Figure 2D). Consistent and significant differences in both MAP and ΔMAP were observed between vehicle and vandetanib throughout the 21‐day dosing period and during the 10‐day “off”‐treatment period (Mann‐Whitney U test, *P* < 0.05; Figure 2A‐F). MAP elevations were also sustained throughout the 10‐day “off”‐treatment period, and this was significant compared to vehicle, during days 1‐5 and 5‐10 (Table S1). There was no evidence of tolerance to the effects of vandetanib, at least in terms of blood pressure responses, during the 21‐day treatment period (Figure 2A‐F).

**Figure 3:**
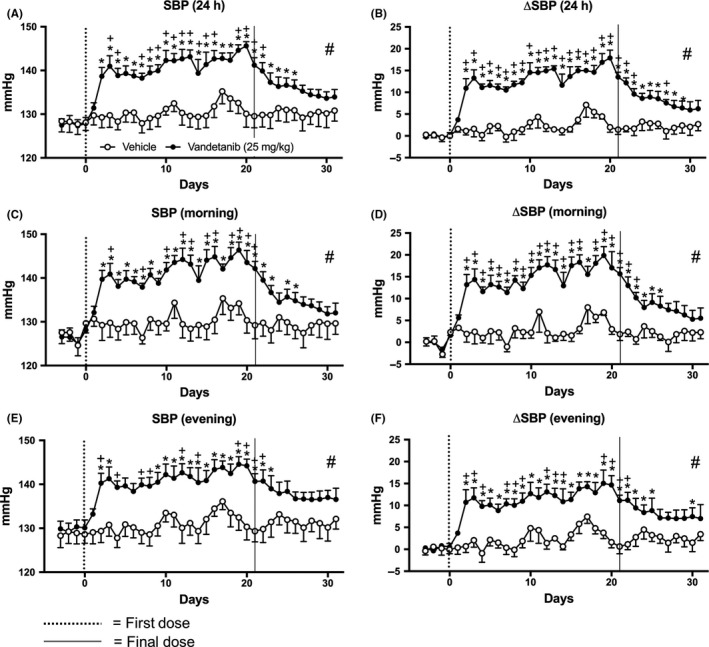
Mean systolic pressure (SBP) of rats dosed with vandetanib 25 mg/kg/day (n = 6) and vehicle (n = 5). A, SBP and B, change in SBP compared to baseline (ΔSBP) measured for 24 hours; C, SBP and D, ΔSBP measured during the morning (06:00‐12:00); E, SBP and F, ΔSBP during the evening (18:00‐23:45). Data displayed as mean ± SEM. *P < 0.05 comparing each time point to baseline; ^+^ *P* < 0.05 comparing vehicle vs vandetanib at the same time point and ^#^ *P* < 0.05 comparing area over or under the curve of vehicle vs vandetanib

**Figure 4:**
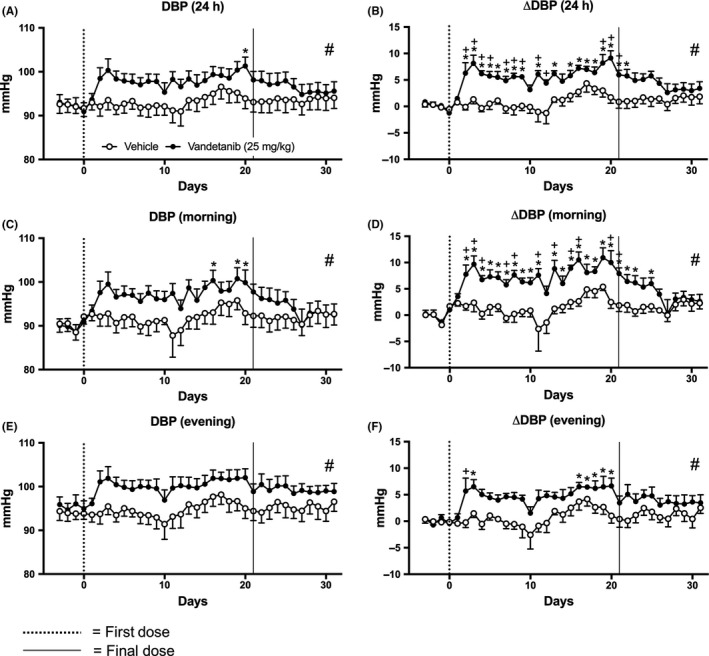
Mean diastolic pressure (DBP) of rats dosed with vandetanib 25 mg/kg/day (n = 6) and vehicle (n = 5). A, DBP and B, change in DBP compared to baseline (ΔDBP) measured for 24 h; C, DBP and D, ΔDBP measured during the morning (06:00‐12:00); E, DBP and F, ΔDBP measured during the evening (18:00‐23:45). Data are displayed as mean ± SEM. *P < 0.05 comparing each time point to baseline; ^+^ *P* < 0.05 comparing vehicle vs vandetanib at the same time point and ^#^ *P* < 0.05 comparing area over or under the curve of vehicle vs vandetanib

Vandetanib significantly (*P* < 0.05) increased SBP and the change in SBP from baseline (ΔSBP) from the second day of dosing (Figure 3A‐F). Vandetanib caused significant increases in ΔSBP compared to vehicle and this elevation appeared to be of a larger magnitude during the morning resting phase (Figure 3D). Significant increases in SBP were also found with vandetanib treatment compared to vehicle for the entire 10‐day “off”‐treatment period (Figure 3A‐F; Table S1). Vandetanib induced smaller increases in mean DBP and change in DBP from baseline (ΔDBP) that is probably not physiologically relevant (Figure 4A‐F; Table S1).

**Figure 5:**
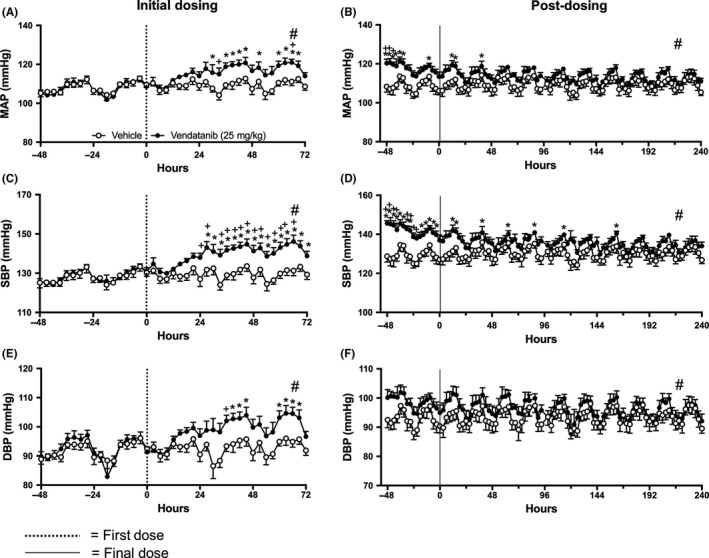
Circadian oscillations of: (A‐B) mean arterial pressure (MAP), (C‐D) systolic blood pressure (SBP), and (E‐F) diastolic blood pressure (DBP) in rats dosed with vandetanib 25 mg/kg/day (n = 6) and vehicle (n = 5). Representing: (A, C, E) 2 days prior to dosing; and the first 3 days of dosing; and (B, D, F) the last 2 days of dosing followed by 10 days “off‐treatment”. Data are displayed as mean ± SEM. *P < 0.05 comparing each time point to baseline; ^+^ *P* < 0.05 comparing vehicle vs vandetanib at the same time point and ^#^ *P* < 0.05 comparing area over or under the curve of vehicle vs vandetanib

Circadian rhythm observations revealed that MAP, SBP, and DBP levels peaked during rodent active evening phase and reached lower levels during the rodent resting phase throughout the baseline and posttreatment periods. During the vandetanib dosing period, these oscillations were disturbed and clear elevations in all three pressure responses were observed (Figure 5A‐F). This contrasted with the better maintained circadian rhythm observed in HR (Figure 1A‐B).

### 3.2 Cardiovascular effects of Pazopanib

#### 3.2.1 Heart rate

Pazopanib did not significantly alter HR compared to baseline (Figure S2a,c,e). As observed with vandetanib above, ΔHR was reduced over time in both the pazopanib and vehicle groups compared to baseline. This was particularly evident during the resting morning phase and this decrease was sustained throughout the 10‐day posttreatment period (Figure S2d). Pazopanib did not significantly alter HR or ΔHR compared to vehicle at any particular time point measured. However, integrated area under the curve analysis revealed significant trend differences between pazopanib and vehicle during the 24 hours, morning and evening measurements of ΔHR (Mann‐Whitney U test, *P* < 0.05; Figure S2b,d,f).

The HR circadian oscillations measured during pazopanib dosing did not differ from vehicle or baseline for first 3 days of treatment (Figure S3a). However, during the 9th and 10th day of the posttreatment period significant tachycardia was found compared to vehicle (Figure S3b). Integrated area under the curve analysis also identified significant trend differences between pazopanib and vehicle throughout the last 2 days of dosing and the 10‐day posttreatment period (Mann‐Whitney U test, *P* < 0.05; Figure S3B). This suggests that HR increases associated with pazopanib only occur post dosing.

#### 3.2.2 Blood pressure

**Figure 6:**
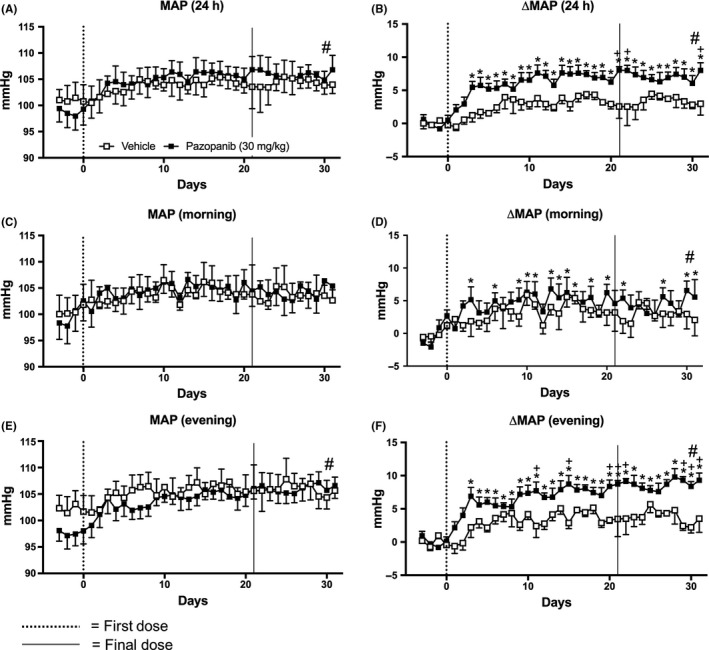
Mean arterial blood pressure (MAP) of rats dosed with pazopanib 30 mg/kg/day (n = 7) and vehicle (n = 4). A, MAP B, change in MAP compared to baseline (ΔMAP) measured for 24 hours, C, MAP and D, ΔMAP measured during the morning (06:00‐12:00), E, MAP and F, ΔMAP measured during the evening (18:00‐23:45). Data displayed as mean ± SEM. *P < 0.05 comparing each time point to baseline; ^+^ *P* < 0.05 comparing vehicle vs pazopanib at the same time point and ^#^ *P* < 0.05 comparing area over or under the curve of vehicle vs pazopanib

Similar to vandetanib, 21 days of pazopanib dosing caused significant pressor effects, when observing ΔMAP, compared to the starting baseline values (Figure 6). However, these effects were not detected by MAP raw data (Figure 6A,C,E). Additionally, when compared to vandetanib treatment, the effects of pazopanib were slower in onset and of smaller magnitude (Figure 6B,D,F). Pazopanib induced significant increases in ΔMAP from the third day of pazopanib dosing, taking 2 days longer to peak compared to the vandetanib‐treated animals (Figure 6B,D,F vs Figure 2B,D,F). This persisted until the end of the experiment (Figure 6B). The effects of pazopanib on MAP during the rodent resting morning phase were variable and somewhat inconsistent (Figure 6D). However, during the active evening period, elevation in ΔMAP was more consistent and sustained throughout both the treatment period (21 days) and “off”‐treatment period (10 days) (Figure 6B,D,F).

**Figure 7:**
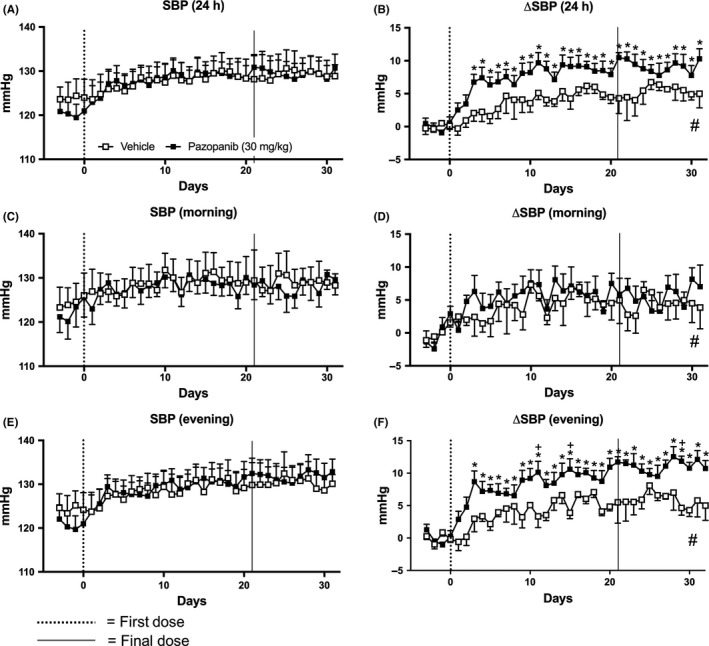
Mean systolic pressure (SBP) of rats dosed with pazopanib 30 mg/kg/day (n = 7) and vehicle (n = 4). A, SBP and B, change in SBP compared to baseline (ΔSBP) measured for 24 hours, C, SBP and D, ΔSBP measured during the morning (06:00‐12:00), E, SBP and F, ΔSBP measured during the evening (18:00‐23:45). Data displayed as mean ± SEM. *P < 0.05 comparing each time point to baseline; ^+^ *P* < 0.05 comparing vehicle vs pazopanib at the same time point and ^#^ *P* < 0.05 comparing area over or under the curve of vehicle vs pazopanib

**Figure 8:**
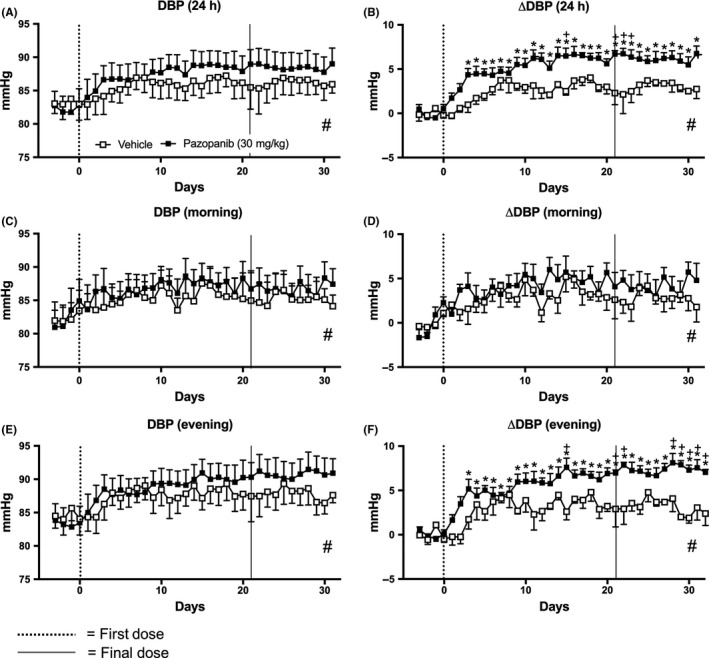
Mean diastolic pressure (DBP) of rats dosed with pazopanib 30 mg/kg/day (n = 7) and vehicle (n = 4). (A) DBP and (B) change in DBP compared to baseline (ΔDBP) measured for 24 hours, (C) DBP and (D) ΔDBP measured during the morning (06:00‐12:00), (E) DBP and (F) ΔDBP measured during the evening (18:00‐23:45). Data displayed as mean ± SEM. *P < 0.05 comparing each time point to baseline; ^+^ *P* < 0.05 comparing vehicle vs pazopanib at the same time point and ^#^ *P* < 0.05 comparing area over or under the curve of vehicle vs pazopanib

Pazopanib significantly increased ΔSBP during the 24 hours and the active evening measurements (Figure 7). This was sustained throughout both the 21‐day dosing period and the 10‐day “off”‐treatment period (*P* < 0.05; Figure 7B,F). However, raw SBP data did not show pazopanib induced changes (Figure 7A,C,E; Table S2). In addition, unlike vandetanib treatment, no changes in ΔSBP were observed in the morning resting phase with pazopanib treatment, compared to baseline. During the active evening phase, there were significant differences between pazopanib and vehicle groups on the 11th and 15th day of the dosing period and the 8th day of the posttreatment period (Figure 7F). Interestingly, integrated area under the curve analysis found trend differences in ΔSBP found between vehicle and pazopanib over the entire experiment, in the 24 hours, morning and evening (Mann‐Whitney U test, *P* < 0.05; Figure 7B,D,F). Similar effects were observed on DBP and ΔDBP (Figure 8).

In contrast to vandetanib, pazopanib did not significantly alter the circadian pattern of MAP, SBP, or DBP during the first 3 days of pazopanib dosing or the last 2 days of pazopanib dosing followed by a 10‐day “off”‐treatment period, compared to vehicle or baseline (Figure S3c‐h).

## 4 DISCUSSION

In the present study, we have demonstrated that, in rats instrumented for radiotelemetry recording of blood pressure and other cardiovascular variables over an extended period, hypertension can be induced by both vandetanib and pazopanib. These findings extend previous telemetry‐based studies in rats using related RTKI treatments.^21^, ^22^, ^23^, ^27^, ^28^ They are also consistent with the hypertensive effects of vandetanib and pazopanib that have been described in the clinical setting^18^; Pinkas et al, 2017). Of particular note in this study, however, is the finding that after extended treatment with both RTKIs, which more closely reflects the clinical regimens, blood pressure remained consistently elevated, even after 8‐10 days following the cessation of treatment. In the case of vandetanib, this time scale is entirely consistent with Phase 1 clinical trials in man that have indicated that this drug is eliminated slowly from the body with a *t*_1/2_ of *circa* 10 days.^29^ Pazopanib is excreted more quickly and the estimated *t*_1/2_ in man is *circa* 30 h (Australian Public Assessment Report PM‐2009‐01084‐4).

The effects of vandetanib were most obvious in terms of elevation in SBP, however, there was also an elevation in DBP, particularly throughout the treatment period. Compared to vandetanib, the increase in blood pressure with pazopanib was slower in onset and smaller in magnitude. The elevation in overall pressure is consistent with our previous studies using these RTKIs in the Doppler flowmetry model, wherein we showed significant increases in MAP with both vandetanib and pazopanib that were associated with vasoconstrictions in the mesenteric and hindquarters vascular beds.^20^ While DBP was not directly measured in these earlier studies, it would be expected that changes in peripheral vascular resistance would strongly affect DBP.^30^ In the present study, it would appear that these RTKIs have directly affected SBP to a greater extent than DBP, likely via mechanisms involving changes in stroke volume and contractility. However, further studies are clearly needed to better understand the effects of RTKIs on DBP and SBP.

It is notable that pazopanib is much more potent as an inhibitor of VEGFR2‐mediated signaling or binding than vandetanib.^5^, ^6^ It is therefore possible that other kinases (other than VEGFR2) may additionally contribute to larger effects of vandetanib on MAP and SBP observed here. These might include RTKs such as EGFR and PDGFR which have higher affinity for vandetanib.^6^

This study is the first to monitor the longer‐term, continuous impact of vandetanib and pazopanib on the cardiovascular system. Similar or related RTKIs such as cediranib,^31^ sorafenib,^20^, ^21^ and sunitinib^28^, ^32^ have been investigated in other studies where the cardiovascular effects of these compounds were monitored over periods of between 4 days and 4 weeks (continuous) treatment, and showed that MAP returns quickly to baseline levels following the end of treatment. Indeed, Blasi et al^32^ noted that the pressor effects of sunitinib diminished even during the last few days of treatment. Moreover, no associated changes in cardiac structure or function were observed.^32^

Observation of the circadian changes in cardiovascular variables revealed that HR, MAP, SBP, and DBP all peaked during the rodent active evening phase and reached lower levels during the rodent resting phase. During the vandetanib dosing period, the oscillations in MAP, SBP, and DBP were disrupted and clear elevations in all three pressure responses were observed. However, during vandetanib treatment the circadian rhythm observed in HR was much better maintained. In contrast, following pazopanib treatment, the periodic circadian variations in all four cardiovascular variables were well maintained. These changes point to multiple factors contributing to the larger and more disruptive changes in diurnal variations in blood pressure observed with vandetanib (relative to pazopanib).

Although the very slow elimination and clearance of vandetanib from the body^29^ is likely to contribute to the maintained increased in blood pressure observed in the present study during the 10‐day “off”‐treatment period, it is also likely that other hemodynamic compensatory mechanisms are contributing to this sustained effect. For example, the diurnal variation in HR is maintained well in the presence of both drugs, although in the case of vandetanib, drug treatment leads to a small bradycardia and a reduction in the peak‐trough amplitudes. During vandetanib treatment, however, maintenance of a regular diurnal change in, particularly, SBP took longer to reestablish after the initial pressure response. It is possible, therefore, that some resetting of the blood pressure occurs during this period. In the case of pazopanib, there are parallel changes in both DBP (elevation) and HR during the drug washout phase that might also point to compensatory changes, particularly since these both appear to result from an extended period of peak blood pressure and HR during the rodent active period.

The mechanisms underlying the hypertensive effects of anti‐VEGF therapies has yet to be fully elucidated, although a reduction in VEGF‐induced nitric oxide (NO) production has been heavily implicated.^33^, ^34^ Under normal physiological conditions, VEGF signaling enhances endothelial‐derived NO production; this vasoactive substance is subsequently available to act on vascular smooth muscle, evoking a vasodilatation and reducing overall peripheral vascular resistance.^33^ Interference with the VEGF signaling cascade decreases the availability of NO, and is associated with vasoconstriction, capillary rarefaction, and consequently hypertension.^33^, ^34^ To date, the pathophysiology of RTKI‐induced hypertension, particularly relating to inhibition of VEGF, is poorly defined. There is some evidence to suggest that concomitant treatment with antihypertensive agents targeting the renin‐angiotensin‐aldosterone system, or calcium signaling pathways, may improve progression‐free survival and overall survival in cancer patients (reviewed by reference.^34^ Moreover, in vitro data suggest that nitrates and beta‐blockers may also be associated with cancer regression. However, until we fully understand the pathways and mechanisms underlying the cardiovascular effects of VEGF which are essential for normal function, future developments in the therapeutic area will remain a challenge.

It is widely acknowledged that VEGF and its receptors are expressed in a variety of tissues under normal physiological conditions.^35^, ^36^, ^37^, ^38^ During development,^39^, ^40^ wound healing^41^ and the luteal phase of the menstrual cycle,^42^ VEGF expression is high. However, its role in the quiescent vasculature has yet to be fully determined and until recently it was believed that VEGF played an insignificant role in the established adult vasculature.

However, there is increasing evidence demonstrating the important role of VEGF in survival of not only angiogenic vessels, but normal, established vasculature as well.^12^, ^35^, ^43^, ^44^ Thus, prolonged anti‐VEGF therapy is associated with the concept of rarefaction, “normalisation” of the vasculature^43^ and remodeling of these newly formed microvessels. This leads to a loss of structural pericyte coverage, and in many cases actual regression of the blood vessels.^12^, ^35^, ^43^ More recently, it has also been suggested that VEGF plays a similar role in maintaining the function of normal well‐established microvessels.^35^ If so, this may explain the hypertension observed in patients following RTKI treatment, since this will naturally lead to an increase in overall total peripheral resistance.^20^, ^44^ If this hypertension is indeed based on structural changes in the vasculature, then this could well contribute to the sustained hypertension observed in the present study during the “off”‐drug period, particularly following vandetanib treatment.

In summary, the present study has demonstrated that the hypertension associated with RTKI treatment can be recapitulated in a conscious rat model and that this cardiovascular effect is substantially maintained during a prolonged “off”‐treatment period. The reasons for this remain to be established, but further work on the potential changes in microvessel structure and the role of local vasoactive substances (eg, NO) should begin to provide important insights into the mechanisms underlying this serious side effect. This is particularly relevant to the clinical situation where antiangiogenic adjunct therapies are being extended from patients with late‐stage cancer to younger patients at an earlier stage of disease progression.

## DISCLOSURES

None declared.

## References

[1] Ferrara N , Adamis AP . Ten years of anti‐vascular endothelial growth factor therapy. Nat Rev Drug Discov. 2016;15(6):385‐403. 10.1038/nrd.2015.17. 26775688

[2] Peach CJ , Mignone VW , Arruda MA , et al. Molecular pharmacology of VEGF‐A isoforms: binding and signalling at VEGFR2. Int J Mol Sci. 2018;19(4):E1264 10.3390/ijms19041264. 29690653 PMC5979509

[3] Koch S , Tugues S , Li X , Gualandi L , Claesson‐Welsh L . Signal transduction by vascular endothelial growth factor receptors. Biochem J. 2011;437(2):169‐183. 10.1042/BJ20110301. 21711246

[4] Shibuya M . Vascular endothelial growth factor (VEGF) and its receptor (VEGFR) signaling in angiogenesis: a crucial target for anti‐ and pro‐angiogenic therapies. Genes Cancer. 2011;2(12):1097‐1105. 10.1177/1947601911423031. 22866201 PMC3411125

[5] Carter JJ , Wheal AJ , Hill SJ , Woolard J . Effects of receptor tyrosine kinase inhibitors on VEGF165a‐ and VEGF165b‐stimulated gene transcription in HEK‐293 cells expressing human VEGFR2. Br J Pharmacol. 2015;172(12):3141‐3150. 10.1111/bph.13116. 25684635 PMC4459029

[6] Davis MI , Hunt JP , Herrgard S , et al. Comprehensive analysis of kinase inhibitor selectivity. Nat Biotechnol. 2011;29(11):1046‐1051. 10.1038/nbt.1990. 22037378

[7] Musumeci F , Radi M , Brullo C , Schenone S . Vascular endothelial growth factor (VEGF) receptors: drugs and new inhibitors. J Med Chem. 2012;55(24):10797‐10822. 10.1021/jm301085w. 23098265

[8] Leboulleux S , Bastholt L , Krause T , et al. Vandetanib in locally advanced or metastatic differentiated thyroid cancer: a randomised, double‐blind, phase 2 trial. Lancet Oncol. 2012;13(9):897‐905. 10.1016/S1470-2045(12)70335-2. 22898678

[9] McCormack PL . Pazopanib: a review of its use in the management of advanced renal cell carcinoma. Drugs. 2014;74(10):1111‐1125. 10.1007/s40265-014-0243-3. 24935162

[10] Ibrahim N , Yu Y , Walsh WR , Yang JL . Molecular targeted therapies for cancer: sorafenib mono‐therapy and its combination with other therapies (review). Oncol Rep. 2012;27(5):1303‐1311. 10.3892/or.2012.1675. 22323095

[11] Le Tourneau C , Raymond E , Faivre S . Sunitinib: a novel tyrosine kinase inhibitor. A brief review of its therapeutic potential in the treatment of renal carcinoma and gastrointestinal stromal tumors (GIST). Ther Clin Risk Manag. 2007;3(2):341‐348. 18360643 10.2147/tcrm.2007.3.2.341 PMC1936316

[12] Carmeliet P , Jain R . Principles and mechanisms of vessel normalization for cancer and other angiogenic diseases. Nat Rev Drug Discov. 2011;10:417‐427. 10.1038/nrd3455. 21629292

[13] Touyz RM , Herrmann SMS , Herrmann J . Vascular toxicities with VEGF inhibitor therapies–focus on hypertension and arterial thrombotic events. J Am Soc Hypertens. 2018;12(6):409‐425. 10.1016/j.jash.2018.03.008. 29703600 PMC6168784

[14] Yang B , Papoian T . Preclinical approaches to assess potential kinase inhibitor‐induced cardiac toxicity: past, present and future. J Appl Toxicol. 2018;38(6):790‐800. 10.1002/jat.3584. 29369373

[15] Shah MA , Ramanathan RK , Ilson DH , et al. Multicenter phase II study of irinotecan, cisplatin, and bevacizumab in patients with metastatic gastric or gastroesophageal junction adenocarcinoma. J Clin Oncol. 2006;24(33):5201‐5206. 10.1200/JCO.2006.08.0887. 17114652

[16] Herrmann J , Yang EH , Iliescu CA , et al. Vascular toxicities of cancer therapies: the old and the new – An evolving avenue. Circulation. 2016;133(13):1272‐1289. 10.1161/CIRCULATIONAHA.115.018347. 27022039 PMC4817363

[17] Wells SA , Robinson BG , Gagel RF , et al. Vandetanib in patients with locally advanced or metastatic medullary thyroid cancer: a randomized, double‐blind phase III trial. J Clin Oncol. 2012;30(2):134‐141. https://doi.org/10.1200%2FJCO.2011.35.5040. 22025146 10.1200/JCO.2011.35.5040 PMC3675689

[18] Hamberg P , Verweij J , Sleijfer S . (Pre‐)clinical pharmacology and activity of pazopanib, a novel multikinase angiogenesis inhibitor. Oncologist. 2010;15(6):539‐547. 10.1634/theoncologist.2009-0274. 20511320 PMC3227994

[19] Pinkhas D , Ho T , Smith S . Assessment of pazopanib‐related hypertension, cardiac dysfunction and identification of clinical risk factors for their development. Cardiooncology. 2017;3 pii: 5. 10.1186/s40959-017-0024-8 . PMC5828231 29497565

[20] Carter JJ , Fretwell LV , Woolard J . Effects of 4 multitargeted receptor tyrosine kinase inhibitors on regional hemodynamics in conscious, freely moving rats. FASEB J. 2017;31(3):1193‐1203. 10.1096/fj.201600749R. 27986807 PMC5295730

[21] Isobe T , Komatsu R , Honda M , Kuramoto S , Shindoh H , Tabo M . Estimating the clinical risk of hypertension from VEGF signal inhibitors by a non‐clinical approach using telemetered rats. Toxicol Sci. 2014;39(2):237‐242. 10.2131/jts.39.237. 24646704

[22] Lankhorst S , Baelde HJ , Kappers MH , et al. Greater sensitivity of blood pressure than renal toxicity to tyrosine kinase receptor inhibition with sunitinib. Hypertension. 2015;66(3):543‐549. 10.1161/HYPERTENSIONAHA.115.05435. 26195484

[23] Collins T , Gray K , Bista M , et al. Quantifying the relationship between inhibition of VEGF receptor 2, drug‐induced blood pressure elevation and hypertension. Br J Pharmacol. 2018;175(4):618‐630. 10.1111/bph.14103. 29161763 PMC5786461

[24] McGrath JC , Drummond GB , McLachlan EM , Kilkenny C , Wainwright CL . Guidelines for reporting experiments involving animals: the ARRIVE guidelines. Br J Pharmacol. 2010;160(7):1573‐1576. 10.1111/j.1476-5381.2010.00873.x. 20649560 PMC2936829

[25] Kramer K , Kinter LB . Evaluation and applications of radiotelemetry in small laboratory animals. Physiol Genomics. 2003;13(3):197‐205. 10.1152/physiolgenomics.00164.2002. 12746464

[26] Sgoifo A , Stilli D , Medici D , Gallo P , Aimi B , Musso E . Electrode positioning for reliable telemetry ECG recordings during social stress in unrestrained rats. Physiol Behav. 1996;60(6):1397‐1401. 10.1016/S0031-9384(96)00228-4. 8946481

[27] Kappers MH , de Beer VJ , Zhou Z , et al. Sunitinib‐induced systemic vasoconstriction in swine is endothelin mediated and does not involve nitric oxide or oxidative stress. Hypertension. 2012;59(1):151‐157. 10.1161/HYPERTENSIONAHA.111.182220. 22124432

[28] Kappers MH , van Esch JH , Sluiter W , Sleijfer S , Danser AH , van den Meiracker AH . Hypertension induced by the tyrosine kinase inhibitor sunitinib is associated with increased circulating endothelin‐1 levels. Hypertension. 2010;56(4):675‐681. 10.1161/HYPERTENSIONAHA.109.149690. 20733093

[29] Martin P , Oliver S , Kennedy SJ , et al. Pharmacokinetics of vandetanib: three phase I studies in healthy subjects. Clin Ther. 2012;34(1):221‐237. 10.1016/j.clinthera.2011.11.011. 22206795

[30] Vlachopoulos C , O'Rourke M . Diastolic pressure, systolic pressure, or pulse pressure? Curr Hypertens Rep. 2000;2(3):271‐279. 10981160 10.1007/s11906-000-0010-6

[31] Curwen JO , Musgrove HL , Kendrew J , Richmond GH , Ogilvie DJ , Wedge SR . Inhibition of vascular endothelial growth factor‐a signaling induces hypertension: examining the effect of cediranib (recentin; AZD2171) treatment on blood pressure in rat and the use of concomitant antihypertensive therapy. Clin Cancer Res. 2008;14(10):3124‐3131. 10.1158/1078-0432.CCR-07-4783. 18483380

[32] Blasi E , Heyen J , Patyna S , et al. Sunitinib, a receptor tyrosine kinase inhibitor, increases blood pressure in rats without associated changes in cardiac structure and function. Cardiovasc Ther. 2012;30(5):287‐294. 10.1111/j.1755-5922.2011.00278.x. 21884012

[33] Facemire CS , Nixon AB , Griffiths R , Hurwitz H , Coffman TM . Vascular endothelial growth factor receptor 2 controls blood pressure by regulating nitric oxide synthase expression. Hypertension. 2009;54(3):652‐658. 10.1161/HYPERTENSIONAHA.109.129973. 19652084 PMC2746822

[34] Agarwal M , Thareja N , Benjamin M , Akhondi A , Mitchell GD . Tyrosine kinase inhibitor‐induced hypertension. Curr Oncol Rep. 2018;20(8):65 10.1007/s11912-018-0708-8. 29931399

[35] Baffert F , Le T , Sennino B , et al. Cellular changes in normal blood capillaries undergoing regression after inhibition of VEGF signaling. Am J Phys Heart Circ Phys. 2006;290:H547‐H559. 10.1152/ajpheart.00616.2005. 16172161

[36] Berse B , Brown LF , Van de Water L , Dvorak HF , Senger DR . Vascular permeability factor (vascular endothelial growth factor) gene is expressed differentially in normal tissues, macrophages, and tumors. Mol Biol Cell. 1992;3(2):211‐220. 10.1091/mbc.3.2.211. 1550962 PMC275520

[37] Kim I , Ryan AM , Rohan R , et al. Constitutive expression of VEGF, VEGFR‐1, and VEGFR‐2 in normal eyes. Invest Ophthalmol Vis Sci. 1999;40(9):2115‐2121. 10440268

[38] Ng YS , Rohan R , Sunday ME , Demello DE , D'Amore PA . Differential expression of VEGF isoforms in mouse during development and in the adult. Dev Dyn. 2001;220(2):112‐121. 10.1002/1097-0177(2000)9999:9999<:AID-DVDY1093>3.0.CO;2-D. 11169844

[39] Carmeliet P , Ferreira V , Breier G , et al. Abnormal blood vessel development and lethality in embryos lacking a single VEGF allele. Nature. 1996;380(6573):435‐439. 10.1038/380435a0. 8602241

[40] Ferrara N , Carver‐Moore K , Chen H , et al. Heterozygous embryonic lethality induced by targeted inactivation of the VEGF gene. Nature. 1996;380(6573):439‐442. 10.1038/380439a0. 8602242

[41] Bao P , Kodra A , Tomic‐Canic M , Golinko MS , Ehrlich HP , Brem H . The role of vascular endothelial growth factor in wound healing. J Surg Res. 2009;153(2):347‐358. 10.1016/j.jss.2008.04.023. 19027922 PMC2728016

[42] Endo T , Kitajima Y , Nishikawa A , Manase K , Shibuya M , Kudo R . Cyclic changes in expression of mRNA of vascular endothelial growth factor, its receptors Flt‐1 and KDR/Flk‐1, and Ets‐1 in human corpora lutea. Fertil Steril. 2001;76(4):762‐768. 10.1016/S0015-0282(01)02012-X. 11591411

[43] Jain RK . Normalization of tumor vasculature: an emerging concept in antiangiogenic therapy. Science. 2005;307(5706):58‐62. 10.1126/science.1104819. 15637262

[44] Mourad JJ , Levy BI . Mechanims of antiangiogenic‐induced arterial hypertension. Curr Hypertens Rep. 2011;13(4):289‐293. 10.1007/s11906-011-0206-y. 21479992

