# Supplementary material for: Long‐term cardiovascular effects of vandetanib and pazopanib in normotensive rats
Source: Pharmacol Res Perspect. 2019 May 31;7(3):e00477. doi: 10.1002/prp2.477 (PMC6543457; doi:10.1002/prp2.477)
Supplement: Supplementary file 1 [file PRP2-7-e00477-s001.pdf]

Supplementary Figures and Tables

Long-term cardiovascular effects of vandetanib and pazopanib in normotensive rats

Samantha L. Cooper [1,2,*], Joanne J. Carter[1,*], Julie March[1], Jeanette Woolard[1,2]

[1]Division of Physiology, Pharmacology and Neuroscience, School of Life Sciences, Queen's Medical Centre, University of Nottingham, Nottingham NG7 2UH, UK
[2]Centre of Membrane Proteins and Receptors (COMPARE), University of Birmingham and University of Nottingham, Midlands NG7 2UH, UK

Supplementary Data
Figure 1

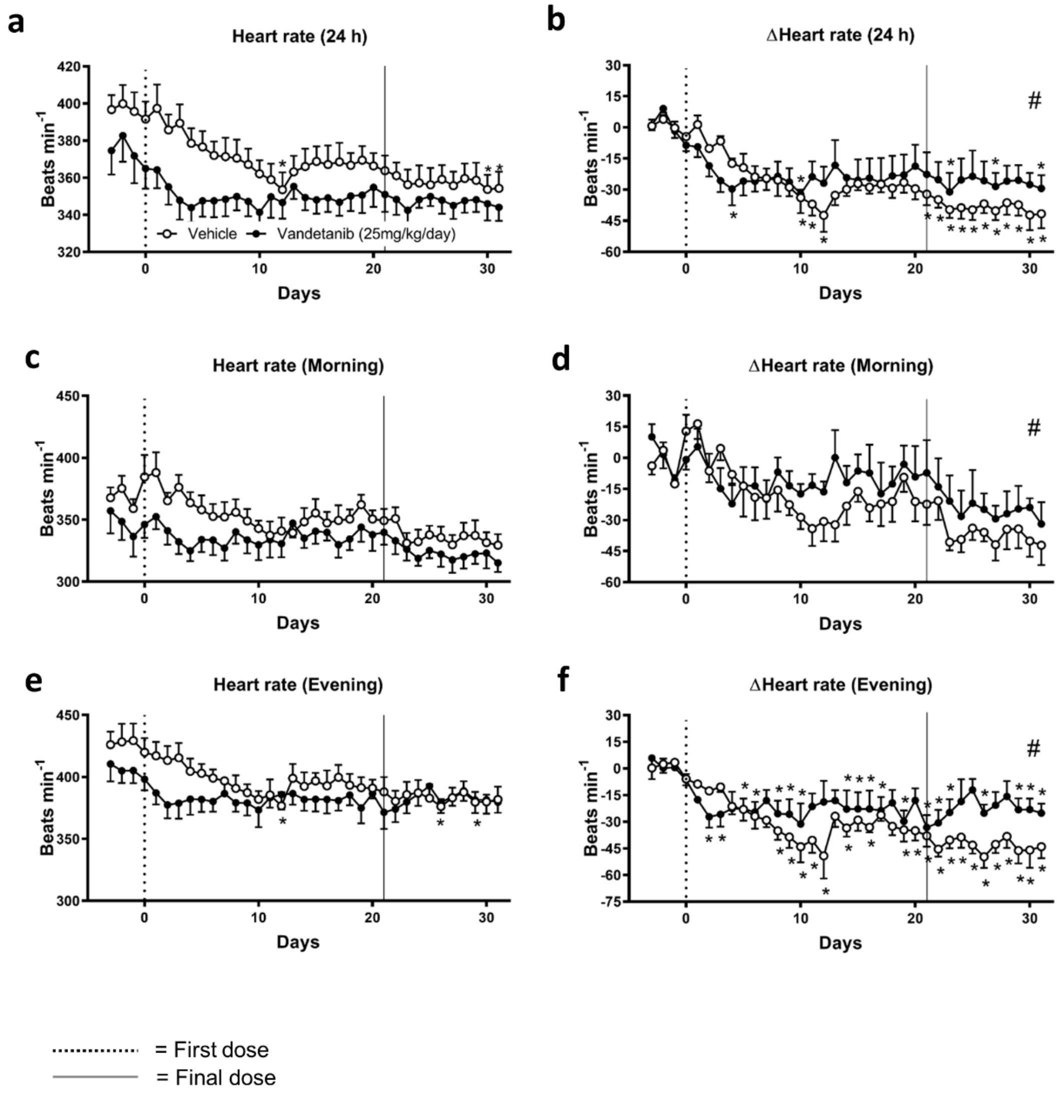

Supplementary Figure 1. Mean heart rate (HR) of rats dosed with vandetanib 25 mg/kg/day (n = 6) and vehicle (n = 5). (a) HR and (b) change in HR compared to baseline (ΔHR) measured for 24 h; (c) HR and (d) ΔHR measured during the morning (06:00-12:00); (e) HR and (f) ΔHR measured during the evening (18:00-23:45). Data are displayed as mean ± SEM. * $p < 0.05$ comparing each time point to baseline; $^{+}$ $p < 0.05$ comparing vehicle vs vandetanib at the same time point and $^{\#}$ $p < 0.05$ comparing area over or under the curve of vehicle vs vandetanib.

Supplementary Data
Figure 2

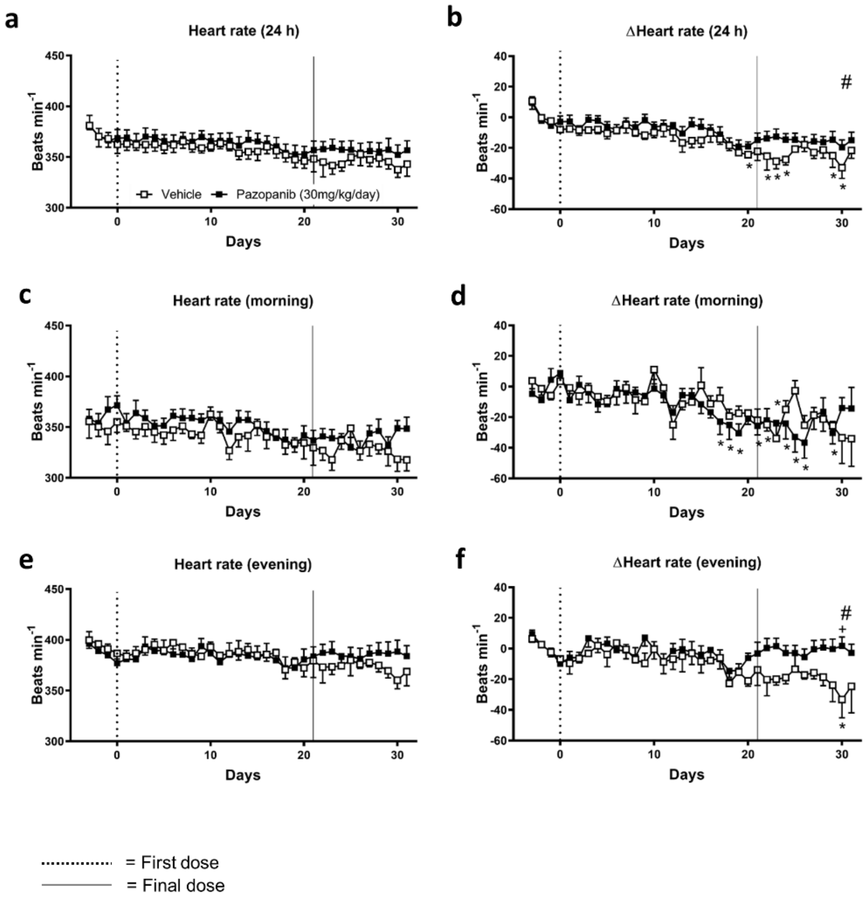

Supplementary Figure 2. Mean heart rate (HR) of rats dosed with pazopanib 30 mg/kg/day (n = 7) and vehicle (n = 4). (a) HR and (b) change in HR compared to baseline (ΔHR) measured for 24 h; (c) HR and (d) ΔHR measured during the morning (06:00-12:00); (e) HR and (f) ΔHR measured during the evening (18:00-23:45). Data are displayed as mean ± SEM. * $p < 0.05$ comparing each time point to baseline; $^{+}$ $p < 0.05$ comparing vehicle vs pazopanib at the same time point and $^{\#}$ $p < 0.05$ comparing area over or under the curve of vehicle vs pazopanib.

Supplementary Data
Figure 3

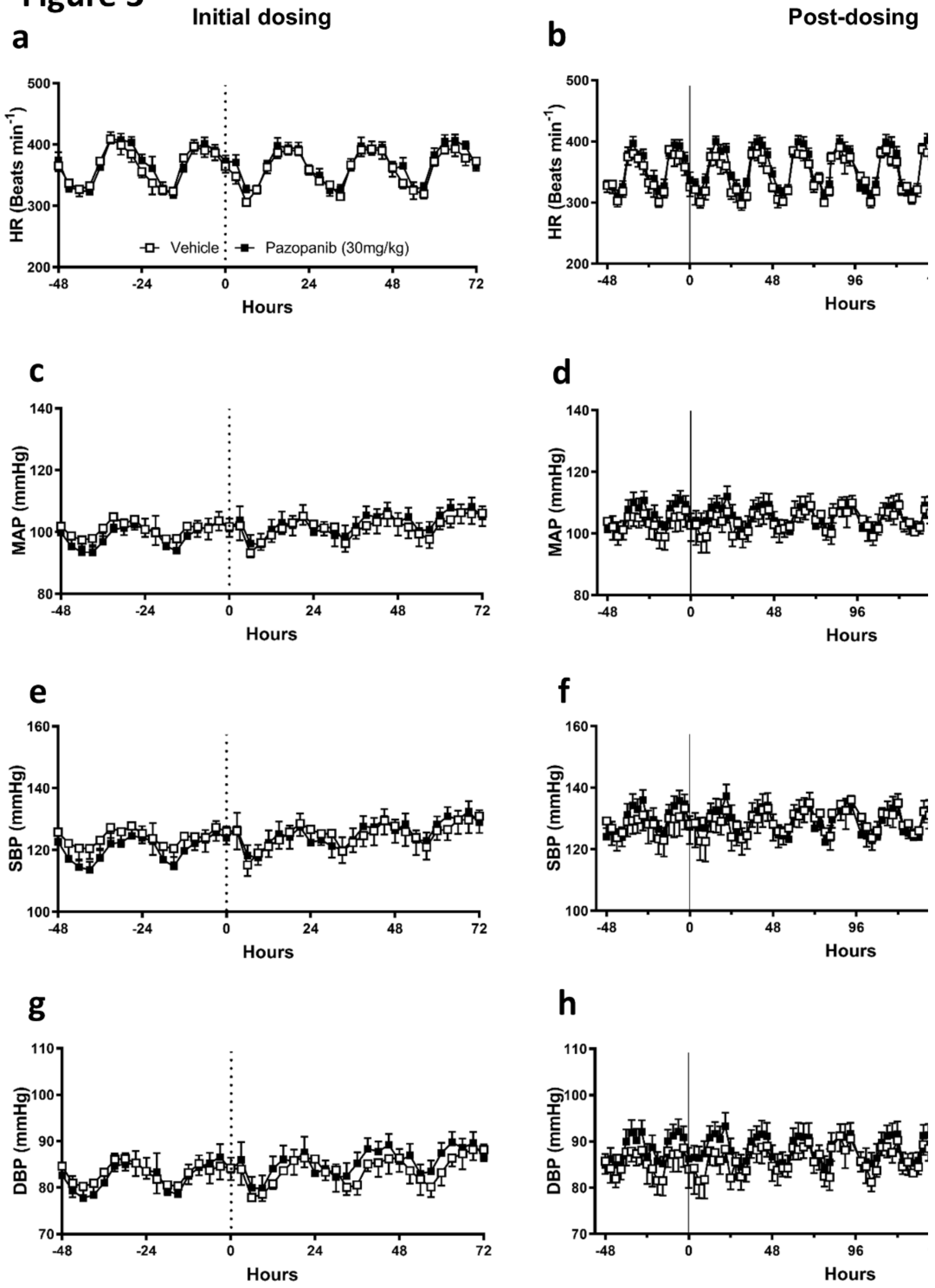

Supplementary Figure 3. Circadian oscillations of: (a-b) HR, (c-d) MAP, (e-f) SBP and (g-h) DBP in rats dosed with pazopanib 30 mg/kg/day (n = 7) and vehicle (n = 4). Representing: (a, c, e, g) 2 days prior to dosing and the first 3 days of dosing; and (b, d, f, h) the last 2 days of dosing followed by 10 days “off-treatment”. Data displayed as mean ± SEM. * $p < 0.05$ comparing each time point to baseline; $^{+}$ $p < 0.05$ comparing vehicle vs pazopanib at the same time point and $^{\#}$ $p < 0.05$ comparing area over or under the curve of vehicle vs pazopanib.

	HR (beats/min)	MAP (mmHg)	SBP (mmHg)	DBP (mmHg)
**1-5 days post-treatment:**				
Vehicle 1 (n=5)	358 ± 1	110 ± 0	131 ± 0	94 ± 0
Vandetanib 25 mg/kg/day (n=6)	347 ± 1*	115 ± 0*	137 ± 1*	97 ± 0*
**5-10 days post-treatment:**				
Vehicle 2 (n=4)	356 ± 1	109 ± 0	130 ± 0	94 ± 0
Vandetanib 25 mg/kg/day (n=6)	346 ± 1*	112 ± 0*	134 ± 0*	95 ± 0*

Supplementary Table 1*: Cardiovascular variables for HR, MAP, SBP and DBP (24 h) measured after cessation of vandetanib (25 mg/kg/day) treatment. Statistics: * = P < 0.05; comparing vehicle to vandetanib treatment.*

	HR (beats/min)	MAP (mmHg)	SBP (mmHg)	DBP (mmHg)
**1-5 days post-treatment:**				
Vehicle 1 (n=5)	346 ± 2	104 ± 0	129 ± 1	86 ± 0
Pazopanib 30 mg/kg/day (n=6)	357 ± 1*	105 ± 0	129 ± 0	88 ± 0*
**5-10 days post-treatment:**				
Vehicle 2 (n=4)	345 ± 2	104 ± 0	129 ± 0	86 ± 0
Pazopanib 30 mg/kg/day (n=6)	355± 1*	106 ± 0*	130 ± 0	88 ± 0*

Supplementary Table 2*: Cardiovascular variables for HR, MAP, SBP and DBP (24 h) measured post pazopanib (30 mg/kg/day) treatment. Statistics: * = P < 0.05; comparing vehicle to pazopanib treatment.*
